# Thermal Properties and In Vitro Biodegradation of PLA-Mg Filaments for Fused Deposition Modeling

**DOI:** 10.3390/polym15081907

**Published:** 2023-04-16

**Authors:** Adrián Leonés, Valentina Salaris, Ignacio Ramos Aranda, Marcela Lieblich, Daniel López, Laura Peponi

**Affiliations:** 1 Instituto de Ciencia y Tecnología de Polímeros (ICTP-CSIC), Calle Juan de la Cierva 2, 28006 Madrid, Spain; aleones@ictp.csic.es (A.L.); v.salaris@ictp.csic.es (V.S.); daniel.l.g@csic.es (D.L.); 2 Centro Nacional de Investigaciones Metalúrgicas (CENIM-CSIC), 28040 Madrid, Spain; marcela@cenim.csic.es

**Keywords:** PLA, magnesium microparticles, in vitro degradation, 3D filament, 3D printing, additive manufacturing, fused deposition modeling

## Abstract

Additive manufacturing, in particular the fused deposition method, is a quite new interesting technique used to obtain specific 3D objects by depositing layer after layer of material. Generally, commercial filaments can be used in 3D printing. However, the obtention of functional filaments is not so easy to reach. In this work, we obtain filaments based on poly(lactic acid), PLA, reinforced with different amounts of magnesium, Mg, microparticles, using a two-step extrusion process, in order to study how processing can affect the thermal degradation of the filaments; we additionally study their in vitro degradation, with a complete release of Mg microparticles after 84 days in phosphate buffer saline media. Therefore, considering that we want to obtain a functional filament for further 3D printing, the simpler the processing, the better the result in terms of a scalable approach. In our case, we obtain micro-composites via the double-extrusion process without degrading the materials, with good dispersion of the microparticles into the PLA matrix without any chemical or physical modification of the microparticles.

## 1. Introduction

Additive manufacturing is a quite new processing method to create a three-dimensional object by adding materials layer by layer [1]. Among the additive manufacturing methods, fused deposition modeling, FDM, is one of the most studied because of its low cost and the use of traditional thermoplastic polymers such as thermoplastic polyurethane (TPU) [2] poly(lactic acid) and PLA [3]. For instance, PLA is a biodegradable polymer widely used in 3D printing applications such as bone tissue repair [4] or biodegradable scaffolds [5]. Additionally, PLA chains are susceptible to being attacked by hydrolytic reactions, turning into non-toxic molecules with a high enough degradation rate for human body usage [6]. However, PLA shows some technical limitations in terms of poor hardness [7] and low degradation rate [8], which have to be improved in order for it to be used in biomedical applications. Moreover, when working with a 3D printer, a homogenous filament is necessary when it comes to the dimension diameter of the 3D printer extruder, which can be considered the greatest challenge when the use of non-commercial filament is desired. Therefore, the main strategy to obtain PLA-based filaments is mixing PLA matrix with other polymers or with nano/micro fillers, which not only allows for the obtention of PLA-based filaments with enhanced mechanical properties, but also the obtention of additional functional properties such as antioxidant or antimicrobial activities, etc. [9,10,11]. Composites of PLA with other metals have been produced in order to improve PLA properties; for example, the addition of iron powder leads to an increase in tensile strength [12], and the introduction of silver nanoparticles enhances the antimicrobial properties of PLA [13]. However, considering biomedical applications, among the inorganic fillers, magnesium metal, Mg, is an interesting reinforcement since Mg^2+^ ions are essential in metabolism at a biochemical level, such as protein synthesis, muscle function, and the nervous and immune system [14,15]. Additionally, the released Mg^2+^ ions present an extraordinary biological response, promoting the formation of collagen in skin wounds [16]. These advantages offer a promising opportunity for the applicability of Mg as a biomaterial. Moreover, PLA-Mg composites have been already fabricated before through FDM, exhibiting an improvement in the main properties of the polymeric matrix due to the incorporation of Mg particles [17].

However, as the physiological medium is water-based, the reaction of Mg with water has to be taken into account. In particular, Mg reacts with H_2_O molecules, leading to the formation of hydrogen gas, H_2_ (g), and hydroxyl groups, OH^−^, which create two challenges to be overcome for biomedical applications [14,18]. The accumulation of H_2_ (g) bubbles in the human body and variation in pH values can compromise the viability of the material for biomedical applications [14]. As can be found in the literature, some authors have reported the enhancement of PLA mechanical properties by adding Mg in terms of creep strength [19] and compression modulus [20]. In addition, in vitro degradation of these compounds has been studied in phosphate buffer saline, PBS [21], in simulated body fluid, SBF [22], and in keratinocyte basal media, KBM [23], demonstrating that Mg particles in PLA matrix prevent pH changes and improve the degradation rate of PLA [24]. However, no works studying the relation between the thermal properties and the later in vitro degradation properties of PLA-based filaments reinforced with Mg for 3D printing have been published yet. The main objective of research related to new PLA-based filaments for FDM is focused mainly on the correlations between the processing of filaments and their final mechanical properties in terms of elastic modulus, tensile strength, or elongation at break [25,26,27].

Furthermore, to the best of our knowledge, we studied for the first time the in vitro degradation of PLA-Mg filaments previous to the FDM processing step. In particular, in our work, we carried out thermal characterization via DSC and TGA, obtaining the thermal properties of PLA-Mg filaments, thus considering how the two-step extrusion process can affect the thermal degradation of the filaments. Moreover, considering that we want to obtain a functional filament for further 3D printing, the simpler the processing, the better the result in terms of a scalable approach. In our case, we obtained micro-composites using a double-extrusion process without degrading the materials, with good dispersion of the microparticles into the PLA matrix without any chemical or physical modification of the microparticles. Finally, an 84-day study in phosphate buffer saline, PBS, media was also performed in terms of H_2_ release and pH changes to check for potential uses in biomedical applications.

## 2. Materials and Methods

Polylactic acid (PLA3051D), 3% D-lactic acid monomer, molecular weight 14.2 × 10^4^ g·mol^−1^, density 1.24 g·cm^−3^), was supplied by NatureWorks^®^. Magnesium microparticles were supplied by Nitroparis (average size < 100 µm, purity > 99.90%).

Each PLA-Mg formulation was prepared according to the following process. Firstly, the PLA pellets were powdered in an A10 Basic IKA miller using liquid nitrogen to prevent thermal degradation at 25,000 rpm. Then, the corresponding amount of Mg microparticles was mixed with the PLA powder for 45 min at 240 rpm and, finally, the extrusion process was carried out in a Rondol co-rotating twin-screw extruder with a screw diameter of 10 mm and length/diameter ratio of 20, working at 40 rpm, with a residence time of 3 min and temperatures of 180, 190, 190, 165, and 120 °C. The PLA-Mg formulations obtained were pelletized and used as feedstock in the single-screw filament extruder.

Therefore, the filaments were obtained from a 3DEVO filament extruder working at 5.0 rpm, heater 1 at 165 °C, heater 2 at 190 °C, heater 3 at 185 °C, and heater 4 at 170 °C; the fan speed was set at 30%. The PLA-Mg filaments were stored in a dryer to avoid humidity spoilage of the PLA.

Once the PLA-Mg filaments were obtained, morphological characterization was carried out via scanning electron microscopy, SEM, (PHILIPS XL30 Scanning Electron Microscope, Phillips, Eindhoven, The Netherlands). All the samples were previously gold-coated (~5 nm thickness) in a Polaron SC7640 Auto/Manual Sputter (Polaron, Newhaven, East Sussex, UK).

Thermal transitions were studied using differential scanning calorimetry in a DSC Q2000 TA instrument under a nitrogen atmosphere (50 mL·min^−1^). The thermal analysis was programmed as follows: First, heating was performed at 10 °C∙min^−1^ from 0 °C up to 180 °C, obtaining the glass transition temperature (T_g_) that was calculated as the midpoint of the transition, the cold crystallization enthalpy (∆H_cc_), and the melting enthalpy (∆H_m_).

Thermogravimetric analysis, TGA analysis, was performed to study the thermal degradation of the PLA-Mg filaments in a TA-TGA Q500 thermal analyzer. Dynamic TGA experiments were performed under a nitrogen atmosphere (flow rate of 50 mL∙min^−1^). Samples were heated from room temperature to 600 °C at 10 °C·min^−1^. In this case, the maximum degradation temperature (T_max_) was calculated as the peak from the first derivative of the TGA curves, and the onset degradation temperature, T_5%_, was taken at 5% of mass loss.

The degree of crystallinity (X_c_%) was calculated taking the value of crystallization enthalpy of pure crystalline PLA (∆H_m_°) as 93.6 J·g^−1^ and W_f_ as the weight fraction of PLA in the sample [28].

(1) $$X_{c}\%=100\times \left(\frac{{\Delta H}_{m}-{\Delta H}_{cc}}{{\Delta H}_{m}^{0}{}^{\circ}}\right)\times \frac{1}{Wf}$$

XRD measurements were performed using a Bruker D8 Advance instrument with a CuK as source (0.154 nm) and a Detector Vantec1. The scanning range was 5° to 60°, and the step size and count time per step were 0.023851° and 0.5 s, respectively.

Fourier transform infrared spectroscopy, FTIR, measurements were conducted using a Spectrum One FTIR spectrometer (Perkin Elmer instruments). Spectra were obtained in the 4000–400 cm^−1^ region at room temperature in transmission mode with a resolution of 4 cm^−1^.

The in vitro degradation process was studied by immersing samples of 2 mm length of each PLA-Mg filament in the corresponding volume of phosphate-buffered saline solution, PBS, maintaining the ratio 20 mL PBS: 1 cm^2^ surface area. The degradation was studied at different times, taking samples after 7, 14, 21, and 28 days to characterize the first month and after 84 days, corresponding to 3 months. The extraction days are named T_x_, where x indicates the number of the corresponding day. The as-obtained PLA-Mg filaments are considered as time 0, T0, and are used as references. Each week, the PBS solution was renovated. The in vitro degradation process was run in an oven at a constant temperature of 37 ± 1 °C and the amount of H_2_ released was recorded daily for up to 28 days.

The pH evolution was measured every 7 days for 130 days with a pH METER-02 (Homtiky) with an error of ±0.01. The mass of the PLA-Mg filament samples was measured before beginning the immersion in PBS and after each extraction day. Samples were removed from the solution, and their surfaces were dried with a paper towel, after which they were weighed, obtaining the “wet samples weight”. Then, the samples were dried for 2 weeks under vacuum; afterward, their weights were measured, obtaining the “dry samples weight”. Water accumulation was obtained from the difference between the mass of the wet and dried samples. The mass loss was calculated from the difference between the mass of the dried samples and the mass of the initial samples. A precision balance was used to weigh all samples within an error of 0.05 mg. The results are given in mass % with respect to the initial mass for each sample.

(2) $$Water\;uptake\;\%=100\times \left(\frac{Weight\;wet-Weight\;dry}{Weight\;dry}\right)$$

(3) $$Mass\;variation\;\%=100\times \left(\frac{Weight\;dry-Weight\;initial}{Weight\;initial}\right)$$

## 3. Results and Discussion

**Figure 1:**
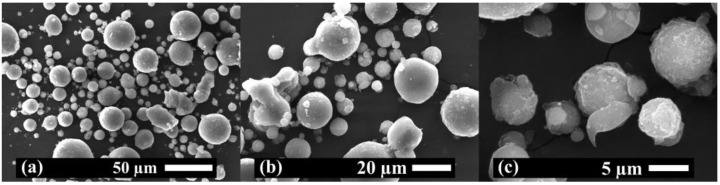
SEM images of Mg microparticles at (**a**) ×500, (**b**) ×1000 and (**c**) ×4000 magnifications.

Previously, to obtain PLA-Mg filaments, a cryogenic mill was used to perform the grinding of Mg powder to Mg microparticles. In Figure 1, SEM images at different magnifications of Mg microparticles are shown to study their average size and morphology. As can be seen, round morphology is observed in Mg microparticles with an average particle size of 12.5 ± 7.4 µm.

**Figure 2:**
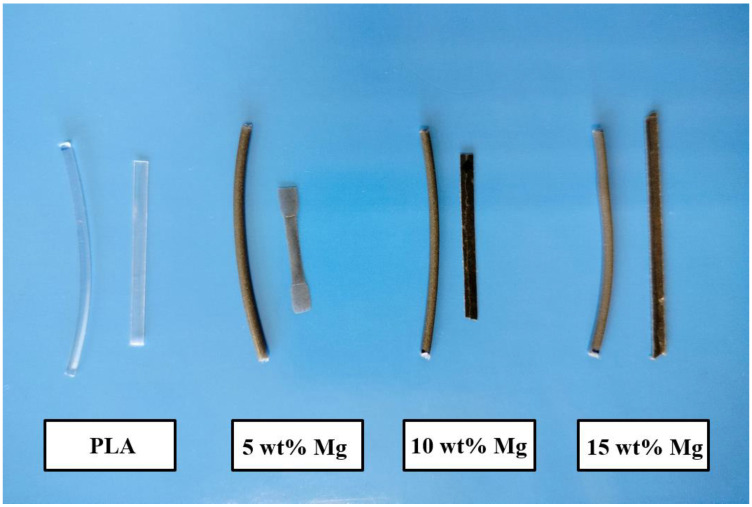
Digital photo of the different filaments and the corresponding 3D printing pieces.

Once the Mg microparticles were characterized, five different Mg-PLA filaments were obtained in a two-extrusion-step process. After the extrusion process, the extruded materials were cut and used as feedstock for a single-screw filament extruder to obtain filament with a diameter of 1.75 mm. In particular, Mg microparticles were added at 1.2, 5, 10, and 15 wt% with respect to PLA. Neat PLA was also extruded and then a filament of PLA was obtained. In Figure 2, a digital photograph of examples of filaments and their corresponding 3D printing pieces is shown.

**Figure 3:**
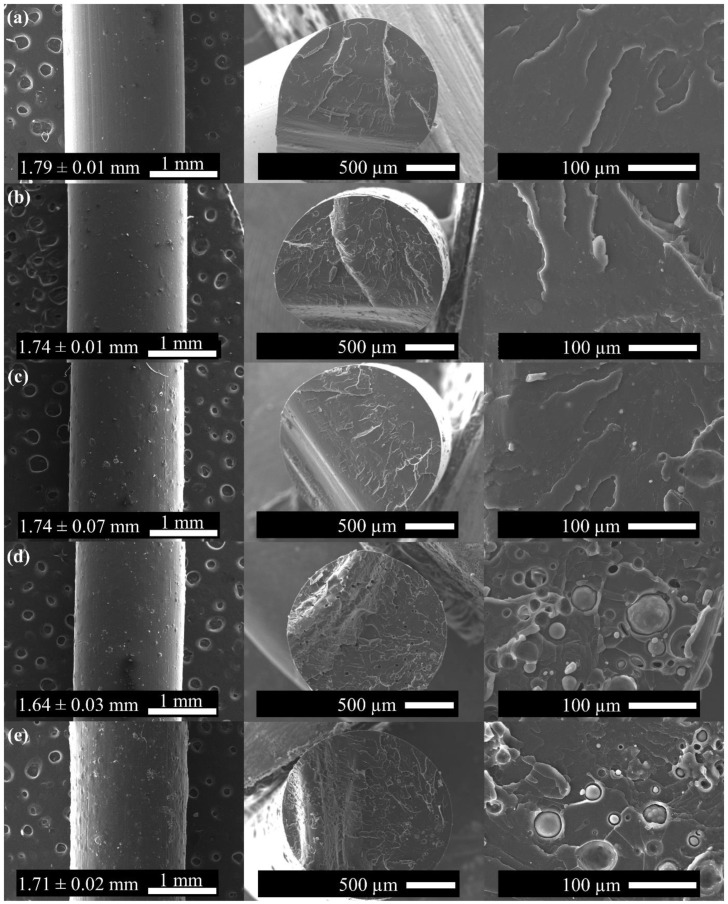
SEM images of (**a**) neat PLA, (**b**) 1.2 wt% Mg, (**c**) 5 wt% Mg, (**d**) 10 wt% Mg, and (**e**) 15 wt% Mg filaments and their fracture surfaces.

To analyze the dispersion of Mg microparticles through the PLA filaments, SEM images were taken both on the filament surface and the fracture surface and are reported in Figure 3. Moreover, the average diameter values of the PLA-Mg filaments are also reported in Figure 3. As can be observed, the neat PLA filament shows smooth morphology with an average diameter of 1.79 ± 0.01 mm, which is in good agreement for its further use in 3D printers. Moreover, as the amount of Mg increases, more irregularities can be observed on the surface of the PLA-Mg filaments. In addition, the average diameters measured for 1.2, 5, 10, and 15 wt% Mg were 1.74 ± 0.01, 1.74 ± 0.07, 1.64 ± 0.03, and 1.71 ± 0.02 mm, respectively. The presence of Mg powder tends to slightly decrease the average diameter of PLA-Mg filaments; however, all values are in the range of diameter values possessed by other commercial filaments. On the other hand, the fracture surface was also studied using SEM analysis. In Figure 3, a fracture pattern characteristic of polymers such as PLA can be observed for the neat PLA filament. According to SEM analysis, in the fracture area, homogeneous distribution of Mg microparticles can be observed through the PLA matrix with good adhesion between the fillers and the matrix.

**Figure 4:**
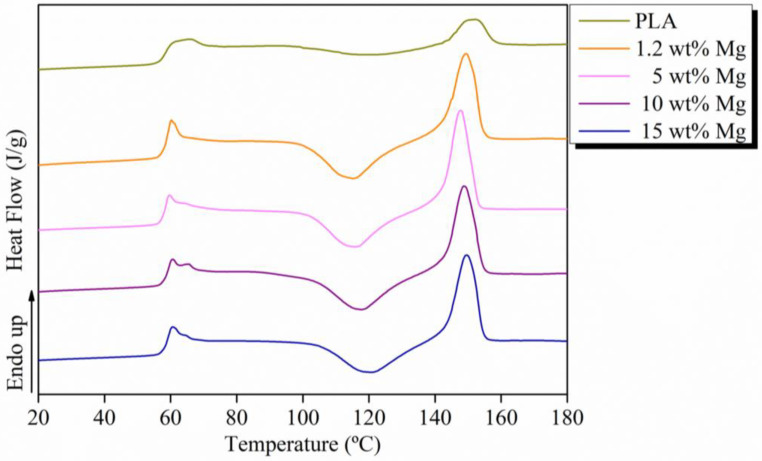
First heating DSC curves for each PLA-Mg filament.

The thermal properties of extruded PLA-Mg filaments were properly obtained from DSC thermograms and are summarized in Figure 4 and Table 1. In particular, the DSC curves show that the glass transition temperature (T_g_) appears at around 60 °C, and is always accompanied by a small endothermic peak due to physical aging, commonly observed in PLA matrices. In addition, the degree of crystallinity remained essentially unchanged, revealing the amorphous structure of PLA-Mg filaments. In particular, X_c_ values of 1.0, 1.0, 0.8, 0.9, and 1.1% were calculated for neat PLA, 1.2, 5, 10, and 15 wt% Mg, respectively. No significant changes in the melting temperatures, T_m_, were observed for any PLA-Mg filaments, with all values being around 150 °C.

**Figure 5:**
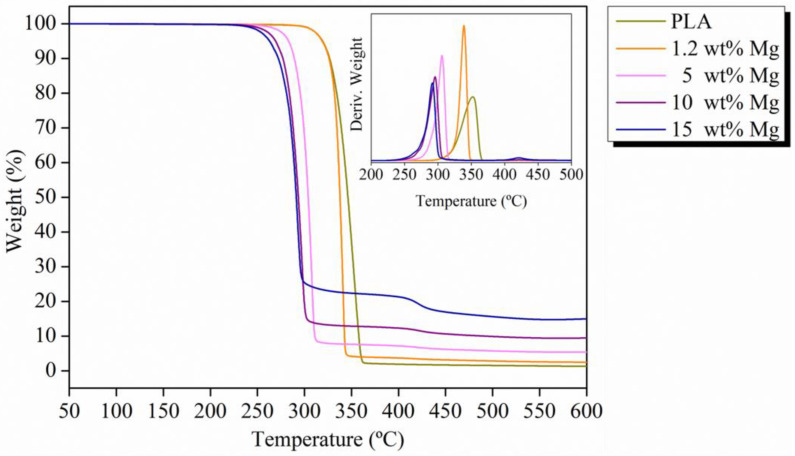
Thermograms and TGA derivate for each PLA-Mg filament.

To evaluate the effect of Mg microparticle addition on the thermal stability and degradation temperatures of the PLA matrix, TGA analysis was carried out on neat PLA and PLA-Mg filaments. As shown in Figure 5 and Table 1, the weight loss curves of Mg-PLA filaments shift towards lower temperatures with respect to neat PLA filament. In particular, the thermal degradation starts at 320 °C for neat PLA and 1.2 Mg wt% filaments, which are the highest temperatures measured, and falls to 263 °C for the highest amount of Mg, 15 wt%. These results show that Mg induces the degradation of PLA. This behavior has been reported in the literature, associated with MgO (present on the surface of Mg particles), which acts as a catalyst for the PLA depolymerization reaction when the polymer is subjected to elevated temperatures [29]. In addition, a second degradation at 425 °C can be observed related to the dehydration of Mg(OH)_2_ on the surface of the Mg microparticles [30]. As can be seen, the residual inorganic amount of each PLA-Mg filament reported in Table 1 corresponds to the Mg wt% calculated, indicating the success of the extrusion protocol. Moreover, considering that the temperature usually used in the FDM process for PLA filaments is around 200 °C [31], our PLA-Mg filaments show suitable thermal stability across the range of Mg concentrations studied for FDM processing.

**Figure 6:**
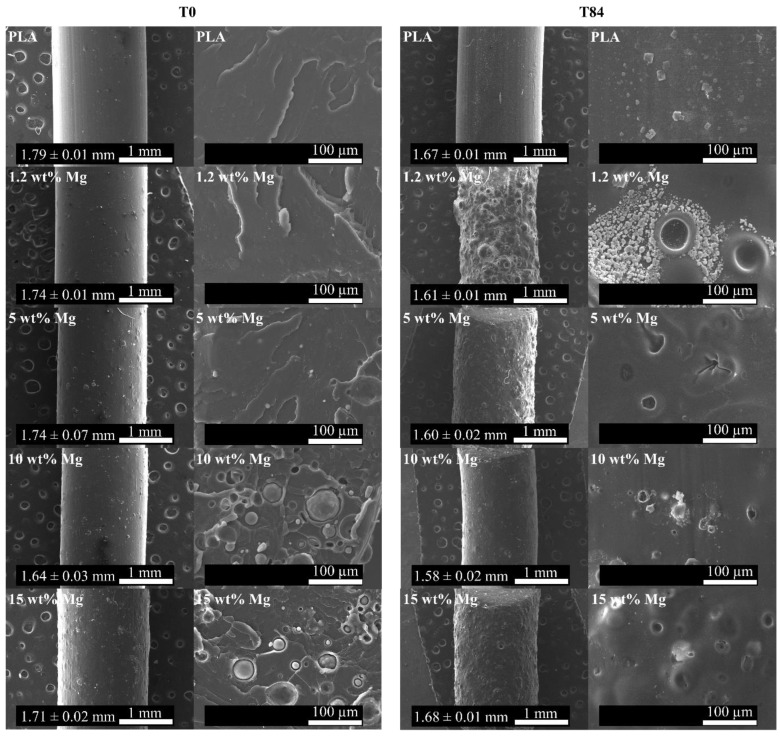
SEM images of PLA-Mg filaments and their fracture surface both at T0 and T84.

Once the thermal characterization of PLA-Mg filaments was carried out, the in vitro degradation test in PBS was performed over the course of 84 days, T84, and compared with the original materials, named T0. To study how the degradation process could affect the morphology, average diameter, and surface of PLA-Mg filaments, fracture surface images at T0 and T84 are shown in Figure 6.

**Figure 7:**
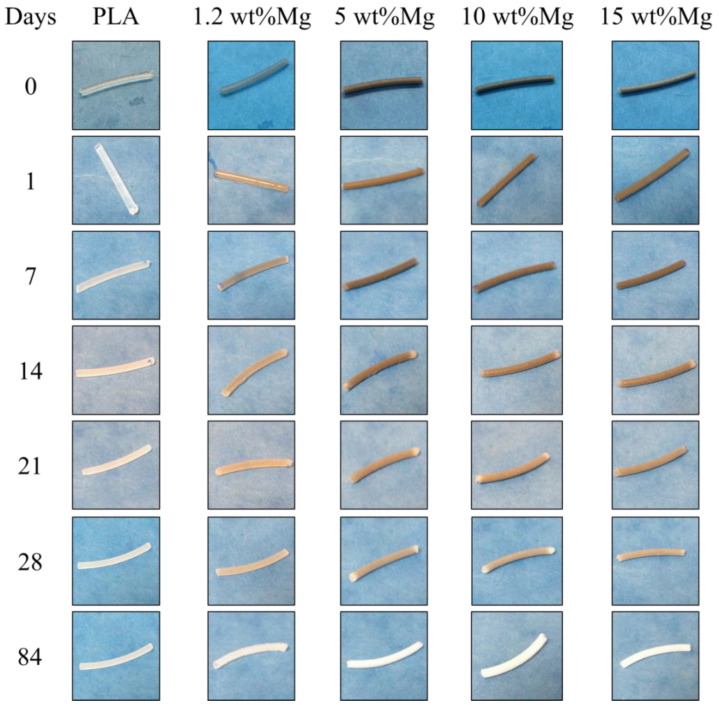
Visual appearance evolution of each PLA-Mg filament at different times.

As can be appreciated, the PLA-Mg filaments after 84 days of immersion in PBS showed an irregular surface with a high number of holes and no presence of Mg microparticles. Remarkably, this phenomenon is observed in the 1.2 wt% Mg filament, which showed the highest eroded surface of all samples. Moreover, the immersion in PBS provoked the precipitation of salts deposited on the surface of the PLA-Mg filaments after 84 days. Parallel to the SEM study, a visual appearance study was carried out to visualize the color changes of the PLA-Mg filaments every seven days, as shown in Figure 7.

Initially at T0, the filaments’ color changed as the amount of Mg increased. In particular, from a white transparent color for neat PLA to soft gray for 1.2 wt% Mg and dark gray for 5, 10, and 15 wt% Mg. As the degradation time increased, a loss of gray color was observed attributed to the release of Mg microparticles, which is in agreement with the previous fracture surface observed via SEM. After 84 days, all PLA-Mg filaments turned white, as was the neat PLA sample.

**Figure 8:**
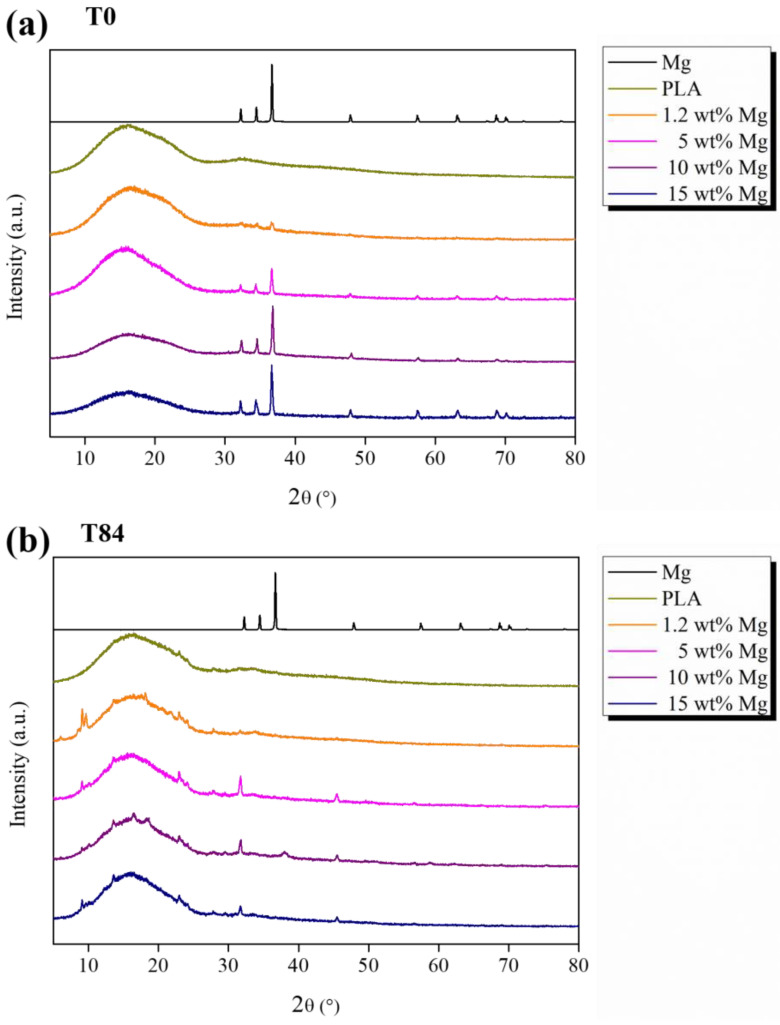
XRD patterns of Mg microparticles as well as PLA-Mg filaments (**a**) at T0 and (**b**) after 84 days, T84, in PBS media.

To corroborate the release of Mg and also study the chemical nature of deposited salts, XRD analysis of PLA-Mg filaments was carried out after 84 days of immersion in PBS and compared with the XRD patterns at T0, shown in Figure 8.

According to XRD patterns, the Mg microparticles show crystallographic peaks located at 2θ = 32.19°, 34.39°, 36.62°, 47.82°, 57.37°, 63.05°, 67.31°, 68.63°, 69.99°, 72.49°, and 77.82° attributed to the [100], [002], [101], [102], [110], [103], [200], [112], [201], [004], and [202] crystallographic planes, respectively [32]. In particular, the presence of Mg microparticles in the Mg-PLA filaments is confirmed by the fact that these peaks can be observed in all XRD patterns shown in Figure 8. However, after 84 days of immersion in PBS, no crystallographic peaks related to Mg were observed, confirming the release of Mg previously discussed. Additionally, the presence of NaCl is confirmed by the peaks observed at 2θ = 32.0°, 45.5°, and 56.5° attributed to the [200], [220], and [222] crystallographic planes of NaCl [32,33].

On the other hand, before starting the degradation test, neat PLA and PLA-Mg filaments show amorphous structure with no crystallographic peaks attributed to PLA, in good agreement with previously Xc (%) values measured using DSC. After 84 days of degradation, the crystallographic peaks located at 16.5°, 19.1°, and 22.5° related to the alpha crystalline phase of PLA [33] were observed for PLA-Mg filaments. As reported in the literature, water molecules showed selective hydrolytic attack towards long PLA chains in the amorphous phase, which highly affects the degree of crystallinity. In fact, the ester groups of aliphatic PLA chains are susceptible to being broken by water molecules, yielding short PLA chains with carboxylate and hydroxyl end groups [33].

**Figure 9:**
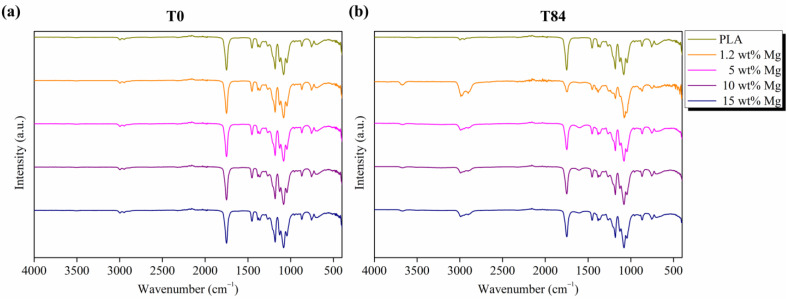
FTIR spectra of PLA-Mg filaments (**a**) at T0 and (**b**) after 84 days in PBS media.

Furthermore, the hydrolysis of ester groups can be monitored using FTIR analysis following the characteristic peaks related to the stretching of the amorphous carbonyl group at 1750 cm^−1^ [33], the stretching of carbonyl in the carboxylate group at 1600 cm^−1^ [33], and the stretching peaks related to O-H groups at 3690 cm^−1^ [33]. With this aim, FTIR analysis was performed on neat PLA and PLA-Mg filaments at T0 and T84 and reported in Figure 9.

It is important to remark that characteristic bands attributed to PLA chemical group vibrations can be observed in all spectra at T0, in particular between 2800 and 3200 cm^−1^, the bands attributed to the symmetrical and asymmetrical stretching vibration of CH groups, and between 1000 and 1500 cm^−1^, the bands attributed to the symmetrical and asymmetrical stretching vibration of C-O-C groups. Moreover, at 830–960 cm^−1^ and 1430–1520 cm^−1^, the bands assigned to the stretching vibration and bending vibrations of CH_3_, respectively, can be observed [33].

It is worth noting that the hydrolytic degradation of the PLA chains was accelerated by the Mg microparticles. Neat PLA filament showed the same characteristic bands at T0 and T84. However, for PLA-Mg filaments, the hydrolytic degradation after 84 days in PBS media was confirmed using FTIR following the intensity of the 1750 cm^−1^ characteristic band attributed to the amorphous carbonyl group at T84, which is slightly lower than at T0. Additionally, the presence of two new bands at 3690 and 1600 cm^−1^, attributed to the stretching of O-H groups and the stretching of carbonyl in the carboxylate group, respectively, can be observed in Figure 9. This acceleration can be explained since Mg is highly hydrophilic, which increases the presence of water molecules that degrade the PLA matrix.

**Figure 10:**
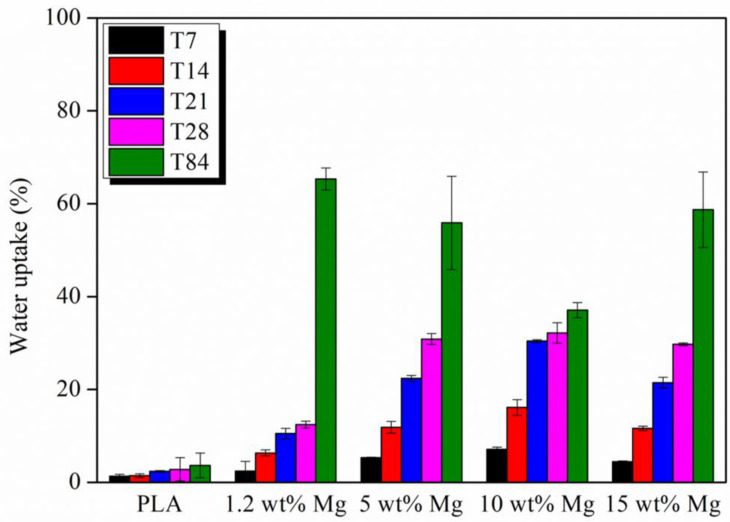
Percentage of water uptake for each PLA-Mg filament after 7, 14, 21, 28, and 84 days in PBS media.

As the degradation rate of PLA is deeply affected by the amount of water, the percentage of water uptake of PLA-Mg filaments during the in vitro degradation test was calculated every 7 days up to 84 days and reported in Figure 10.

The hydrolysis of PLA is controlled by the diffusion of water molecules in the amorphous phase [34]. As previously proposed, the presence of Mg highly increases the water uptake, which implies an increase in the water diffusion through the PLA matrix as seen in Figure 10. It is important to remark that after 84 days, the amount of water is almost constant in all PLA-Mg filaments, no matter the amount of Mg. As previously discussed, after 84 days of immersion in PBS, the Mg released provoked a high number of holes and erased surfaced sites where water molecules can degrade the PLA matrix.

The main concern in the application of Mg in the biomedical field is its reaction with water, as shown in Equation (4), in particular the generation of hydrogen, H_2_ [14].

(4) $$Mg+2H_{2}O\to {Mg}^{2+}+2\left({OH}^{-}\right)+H_{2}$$

**Figure 11:**
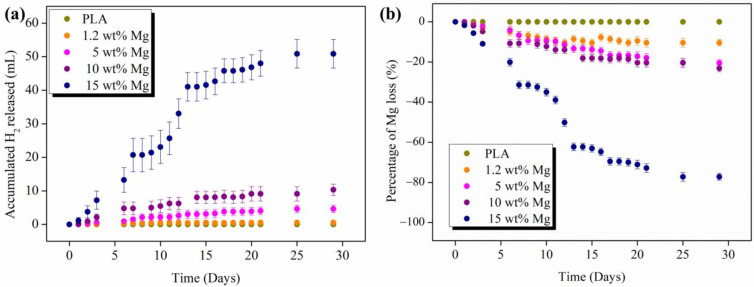
(**a**) Accumulated amount of hydrogen released as a function of immersion time in PBS and (**b**) Mg loss in terms of percentage of mass loss with respect to the Mg initial weight as a function of immersion time in PBS.

The problem of H_2_ released in the human body cannot be explained in terms of how much hydrogen is produced but instead has to be explained in terms of time. In Figure 11a, the H_2_ release as a function of immersion time can be observed.

The accumulated H_2_ released depends on the Mg content of the PLA-Mg filaments. As expected, with higher Mg content, the hydrogen release rate increases up to a volume of 50.9 mL of H_2_ for the highest amount of 15 wt% Mg after 28 days in PBS. It is important to remark that in any case, the maximum amount of H_2_ tolerated by the human body, (2.25 mL/cm^2^/day) [14], is not achieved, which reveals that our materials are suitable for biomedical uses. Moreover, the H_2_ release kinetics start to stabilize after the first two weeks, where a plateau in the accumulated H_2_ released is observed in all PLA-Mg filaments and kept constant during the 28 days. In addition, the amount of Mg that reacted to produce the measured hydrogen was stoichiometrically calculated and is shown in Figure 11b as a percentage of Mg mass loss with respect to the initial Mg weight in each filament. It is important to point out that after 28 days, the 15 wt% Mg filament lost more than 70% of the Mg, which is in accordance with the large volume of H_2_ measured for this sample. Moreover, as the amount of initial Mg increases, the percentage of Mg loss increases by increasing the time in PBS media. Considering that these percentages are stoichiometrically calculated by hydrogen measurements, we can approximate that, after 84 days, 100% of Mg will be released at least for the highest amount of 15 wt% Mg, which is in good agreement with the XRD analysis previously discussed.

**Figure 12:**
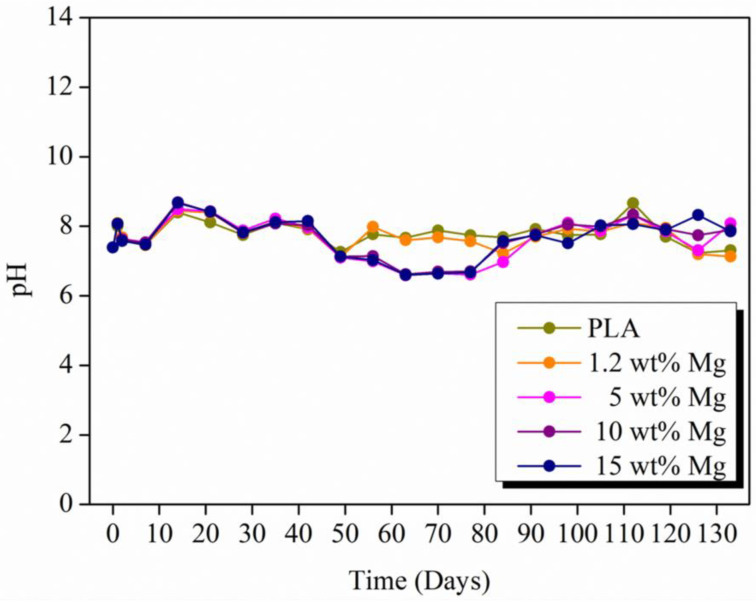
Evolution of pH values for each PLA-Mg filament during degradation in PBS media.

Another concern regarding the Mg reaction with physiological environments is the alkalization of the surrounding area due to the OH^−^ produced. To verify if the buffer capacity of the physiological environment can be surpassed by the alkalization produced in the reaction of Mg with water, the variation in pH in the PBS media was measured every seven days and is reported in Figure 12.

As can be observed, the pH values fluctuated around 7.6 for 5 months for all PLA-Mg filaments. It is important to point out that the pH evolution is quite similar during the first 50 days in PBS media, after which slightly different pH values were observed, in particular from 7.9 for neat PLA to 6.9 for 15 wt% Mg. This is a contradictory result, taking into account that 15 wt% Mg is the material that releases the highest amount of hydrogen, which implies that it must induce the largest increment in pH (alkaline) as well. This behavior may be understood considering that the polymeric matrix can degrade and release lactic acid to the medium, which induces a pH decrement that can compensate for the increment in pH caused by the reaction of Mg.

**Figure 13:**
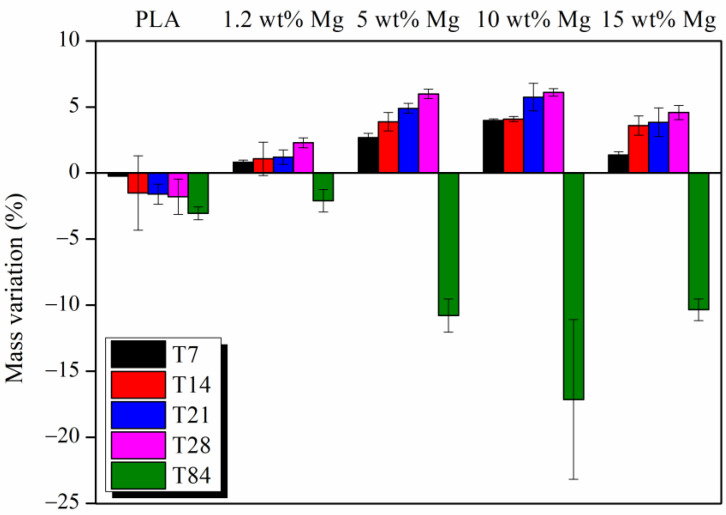
Percentage of mass variation in PLA-Mg filaments after 7, 14, 21, 28, and 84 days in PBS media.

Finally, Figure 13 shows the mass variation of PLA-Mg filaments after 7, 14, 21, 28, and 84 days in PBS media. The PLA-Mg filaments gain mass during the first 28 days, probably due to NaCl precipitation on the surface of the filaments. However, as discussed above, the Mg released induces the formation of pores and holes through the surface of the filaments. Thus, the irregular morphology increases the surface where water molecules can attack the PLA chains. As can be seen in Figure 13, after 84 days, a high loss of mass was measured for all PLA-Mg filaments; remarkably, at this time, T84, the water uptake reported in Figure 10 was the highest. Furthermore, it seems that Mg release occurs after the 28 first days and then the water uptake increases up to the maximum values where the highest PLA degradation rate is observed, losing 3, 10, 15, and 10% of mass for 1.2, 5, 10, and 15 wt% Mg, respectively. On the other hand, the neat PLA filament started to lose mass since the first week in PBS but the PLA degradation was not enough to observe the FTIR bands related to carboxylate and hydroxyl end groups.

## 4. Conclusions

In this work, PLA-Mg filaments for further 3D printing with different Mg microparticle concentrations were successfully obtained. In particular, Mg microparticles were added at 1.2, 5, 10, and 15 wt% with respect to the polymeric matrix, obtaining an average diameter of the filaments in the range of diameters used in 1.75 mm 3D printers. All the extruded PLA-Mg filaments showed a T_g_ around 60 °C and a T_m_ around 150 °C; however, the presence of Mg microparticles does not affect the small degree of crystallinity of pure PLA. Furthermore, PLA-Mg filaments showed high thermal stability, with PLA and 1.2 MgO wt% presenting the highest T_max_ values of 353 and 339 °C, respectively, this value further decreasing down to 291 °C at 15 wt% of Mg. This phenomenon can be related to the presence of Mg and in particular the MgO present on the surface of the microparticles inducing PLA degradation. However, these degradation temperatures are higher than the temperature used in the extrusion process as well as in 3D printing, which is generally 200 °C for PLA-based materials. After the fabrication and characterization of the materials, in vitro degradation in PBS was studied. From a morphological point of view, PLA-Mg filaments present an irregular surface and holes after 84 days due to the degradation process and the release of Mg microparticles. In fact, no presence of Mg microparticles was observed after 84 days through XRD analysis, confirming the release of Mg. Furthermore, all the samples with Mg microparticles presented a high water uptake capacity, which increases water diffusion through the material, improving degradation. The evaluation of H_2_ release at different immersion times revealed that, as expected, 15 wt% of Mg showed the highest volume of H_2_ released, without, however, reaching the maximum amount of H_2_ tolerated by the human body. Thus, taking into account the obtained results, we can conclude that our materials are suitable for potential 3D printed biomedical applications.

## Figures and Tables

**Table 1:** Thermal properties for each PLA-Mg filament obtained from DSC curves.

Sample	T_g_ (°C)	∆H_cc_ (J/g)	T_cc_ (°C)	∆H_m_ (J/g)	T_m_ (°C)	X_c_ (%)	T_5%_ (°C)	T_max_ (°C)	R. Mg wt%*
PLA	60	7.43	118	9.43	153	2.0	320	353	0.0
1.2 wt% Mg	59	22.29	114	25.16	150	3.1	320	339	1.1
5 wt% Mg	59	23.69	116	23.80	148	0.8	283	306	4.8
10 wt% Mg	59	23.34	118	23.48	149	0.9	269	295	9.9
15 wt% Mg	59	20.72	121	21.72	150	1.1	263	291	14.6

R. Mg wt%*: Residual Mg wt% measured at 600 °C via TGA.

## Data Availability

Not applicable.
